# Supplementary material for: Arabidopsis COP1 guides stomatal response in guard cells through pH regulation
Source: Commun Biol. 2024 Feb 5;7:150. doi: 10.1038/s42003-024-05847-w (PMC10844630; doi:10.1038/s42003-024-05847-w)
Supplement: Supplementary file 2 — Supplementary Information [file 42003_2024_5847_MOESM2_ESM.pdf]

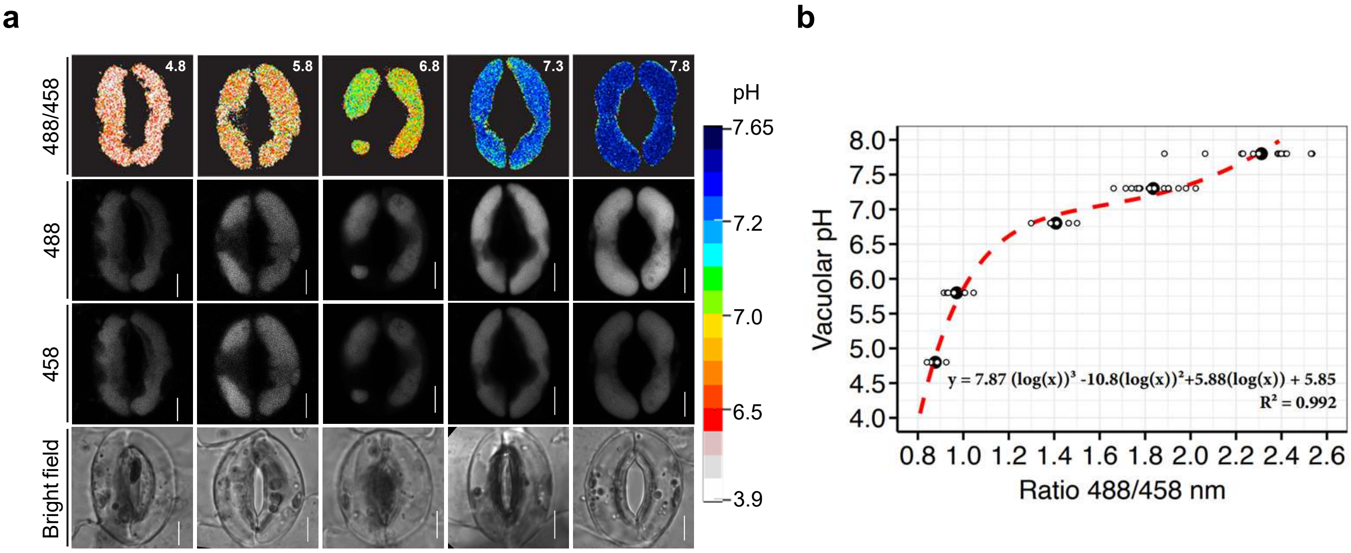

Supplementary Figure 1. ***In vivo*** **BCECF-AM calibration. a** Representative images of four channels (488/458, 488, 458, and bright field) were obtained for wild-type (WT) guard cells after 30 min of incubation in equilibration buffers with pH ranging from 4.3 to 7.8. (Scale bar, 5 µm.) **b** The fluorescence ratios (488/458 nm) from (**a**) were plotted against the pH of the equilibration buffers to obtain a calibration curve. The equation for the calibration curve was derived from log-transformed polynomial regression. Average ratio values were from at least 10 stomata per pH.

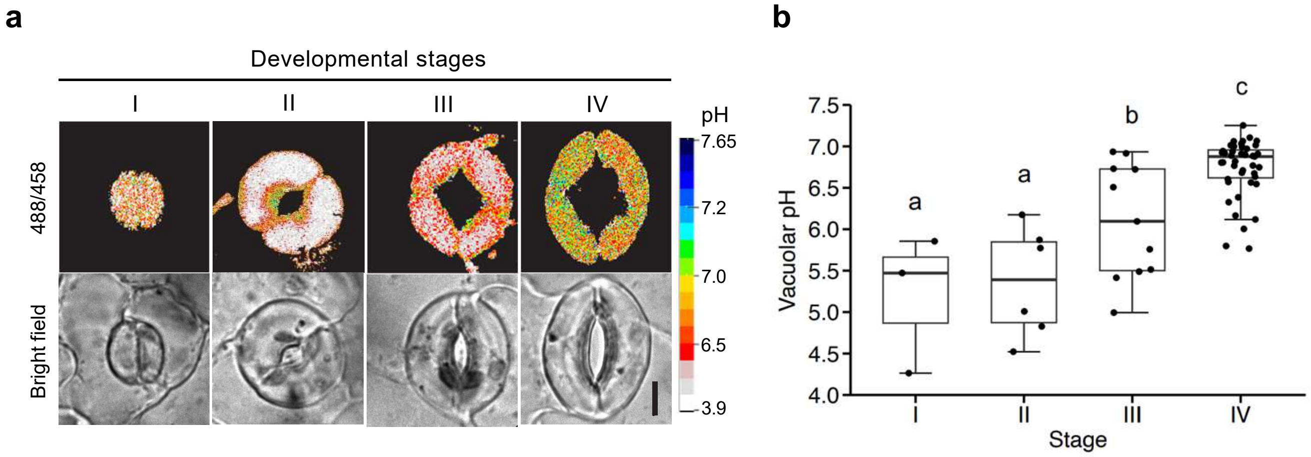

Supplementary Figure 2. Developmental stages-dependent vacuolar pH in guard cells. a Representative pseudocolor ratiometric images of vacuolar pH in guard cells of wild-type (WT) at four different stomatal developmental stages (top) and their corresponding bright field images (bottom). Guard cells were loaded with the pH-sensitive vacuolar loading dye BCECF-AM and subjected to 2 hours of light-induced stomatal opening. Absolute pH calibration was performed using the calibration curve obtained in Supplementary Fig. 1. (Scale bar, 5 µm.) **b** Quantified vacuolar pH at each developmental stage from (**a**). Different letters indicate statistically significant differences between stages ($P < 0.05$); one-way ANOVA; Tukey's HSD.

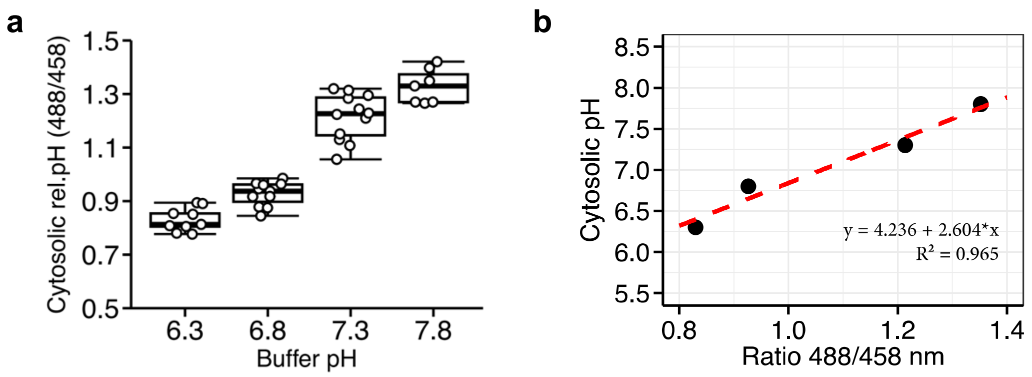

Supplementary Figure 3. *In vivo* ClopHensor calibration. a Guard cells of wild-type (WT) expressing ClopHensor were incubated for 30 min in pH equilibrium buffers with pH 6.3, 6.8, 7.3 and 7.8. Relative cytosolic pH was calculated by dividing the emission images acquired in the 488 nm channel by those obtained in the 458 nm channel. **b** The fluorescence ratios (488/458 nm) from (**a**) were plotted against the pH of the equilibration buffers to obtain a calibration curve. Individual data points are omitted as they were plotted in (**a**). The equation for the calibration curve was derived from linear regression. Average ratio values were from at least 10 stomata per pH.

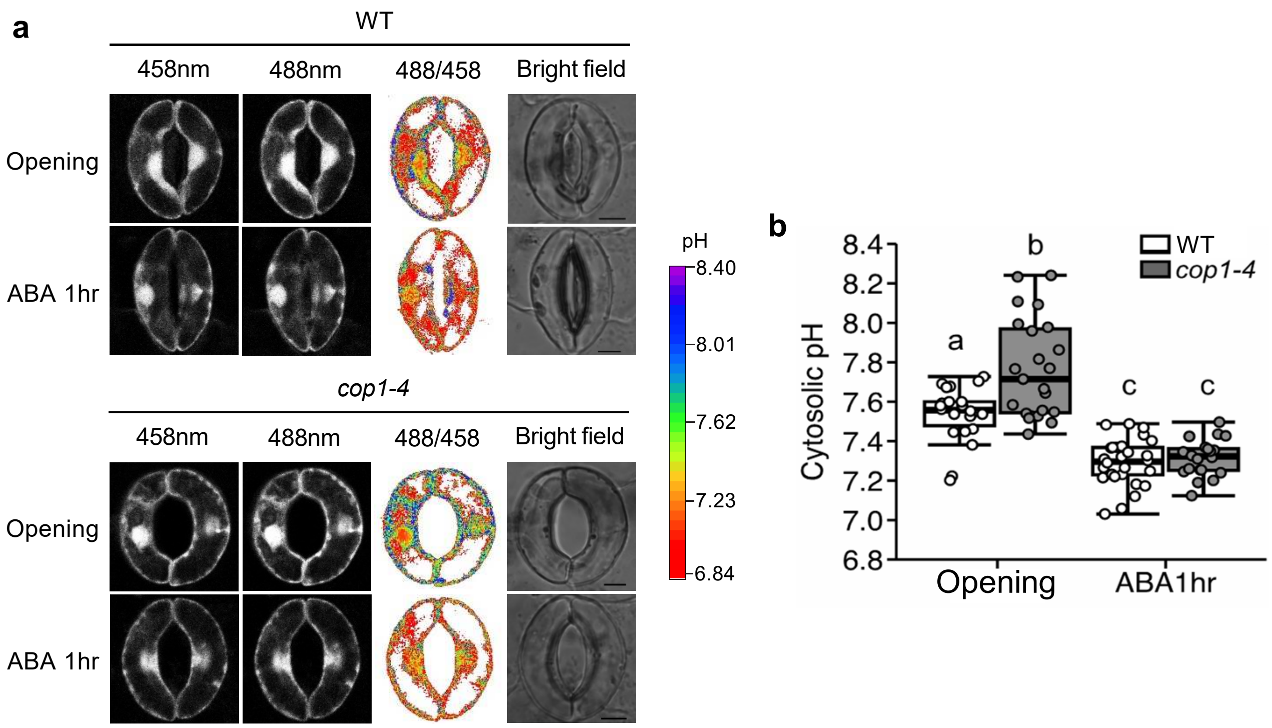

Supplementary Figure 4. Prolonged abscisic acid (ABA) treatment induces cytosolic acidification of wild-type (WT) and *cop1-4* mutant guard cells. a Representative pseudocolor ratiometric images of guard cells of WT and *cop1-4* mutants each expressing ClopHensor under prolonged ABA treatment. Detached leaves were incubated with stomatal opening buffer for 2 hours, and then treated with 10 μM ABA for 1 hour. Fluorescence intensity of cytosolic pH in guard cells of WT and *cop1-4* mutants was measured directly from detached leaves using confocal laser scanning microscope (Leica SP8 X). Pseudocolor ratiometric images were generated by dividing the emission images obtained at 488 nm by those acquired at 458 nm. (Scale bar, 5 μm.) **b** Quantification of ratiometric values for cytosolic pH in guard cells shown in (**a**). Different letters indicate statistically significant differences between the plants ($P < 0.05$); one-way ANOVA; Tukey's HSD.

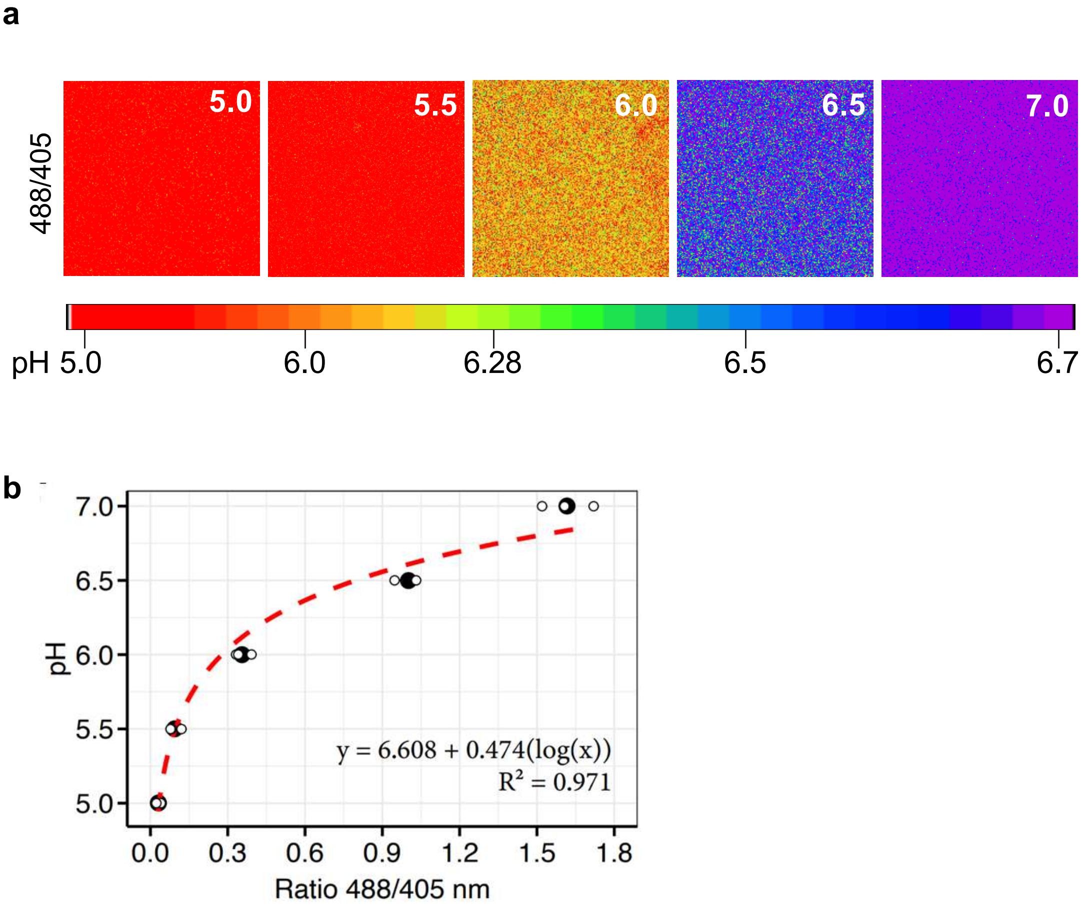

Supplementary Figure 5. *In situ* HPTS calibration. a Representative pseudocolor images of HPTS-stained liquid samples with pH ranging from 5.0 to 7.0. Fluorescence ratios (488/405 nm) were calculated from the emission images obtained in the respective channels. **b** Calibration curve obtained by plotting the fluorescence ratios against the pH of the equilibration buffers. The equation for the calibration curve was derived from log-transformed linear regression. Average ratio values were obtained from at least three images per pH.

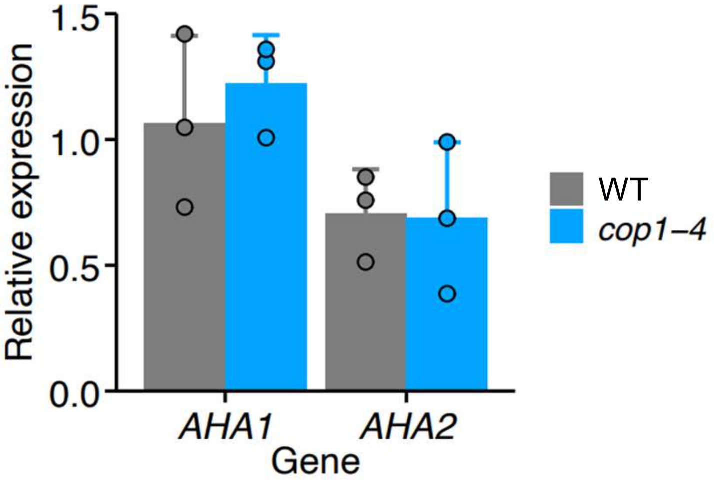

Supplementary Figure 6. Real time-quantitative PCR analysis of *AHA1* and *AHA2* in *cop1-4* mutants. Relative transcript expression levels of PM H^+-ATPases (*AHA1*, *AHA2*) in 14-day-old seedlings of wild-type (WT) and *cop1-4*. The relative expression levels ($2^{-\Delta\Delta Ct}$) were normalized to WT. Mean ± SD are expressed.

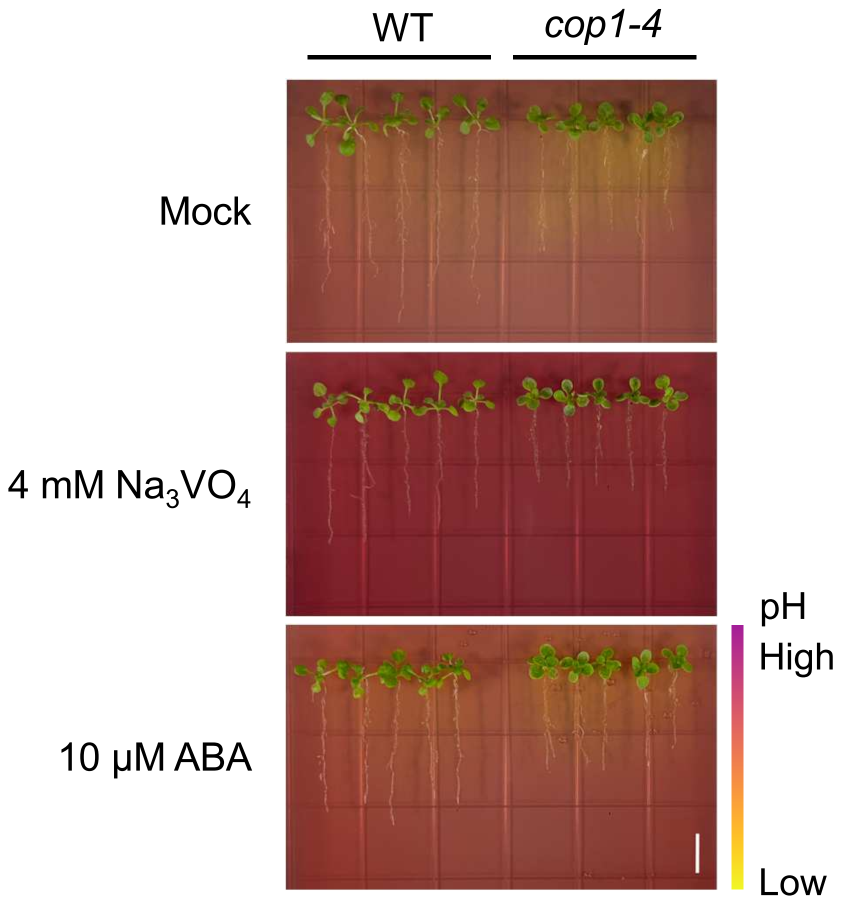

Supplementary Figure 7. Proton extrusion levels of wild-type (WT) and *cop1-4* mutant roots decreased upon abscisic acid (ABA). Rhizosphere acidification assays were conducted using WT and *cop1-4* mutants. Eleven-day-old seedlings were transferred to ½ MS media containing 0.003% of pH indicator dye bromocresol purple in the absence or presence of ABA, and then grown vertically. In order to confirm that the rhizosphere acidification was due to plasma membrane H^+-ATPase activity, another sets of seedlings were transferred to ½ MS media containing both bromocresol purple and 4 mM sodium orthovadanate (Na_3VO_4). Color changes were recorded after 36 hours. (Scale bar, 1 cm.) The experiment was conducted three times, and one representative experiment is shown.

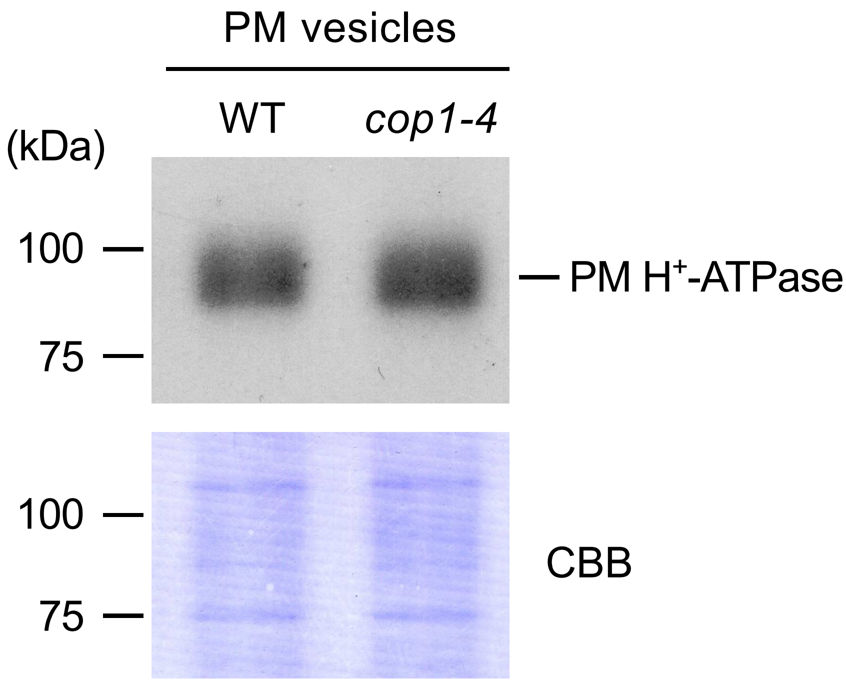

Supplementary Figure 8. Quantification of plasma membrane (PM) H^+-ATPases in isolated PM vesicles from wild-type (WT) and *cop1-4* mutants. PM vesicles from Fig. 4b were subjected to 8% SDS-PAGE, and then the protein levels of PM H^+-ATPases were examined by western blotting with anti-PM H^+-ATPases antibody. Coomassie Brilliant Blue (CBB) was used as a loading control.

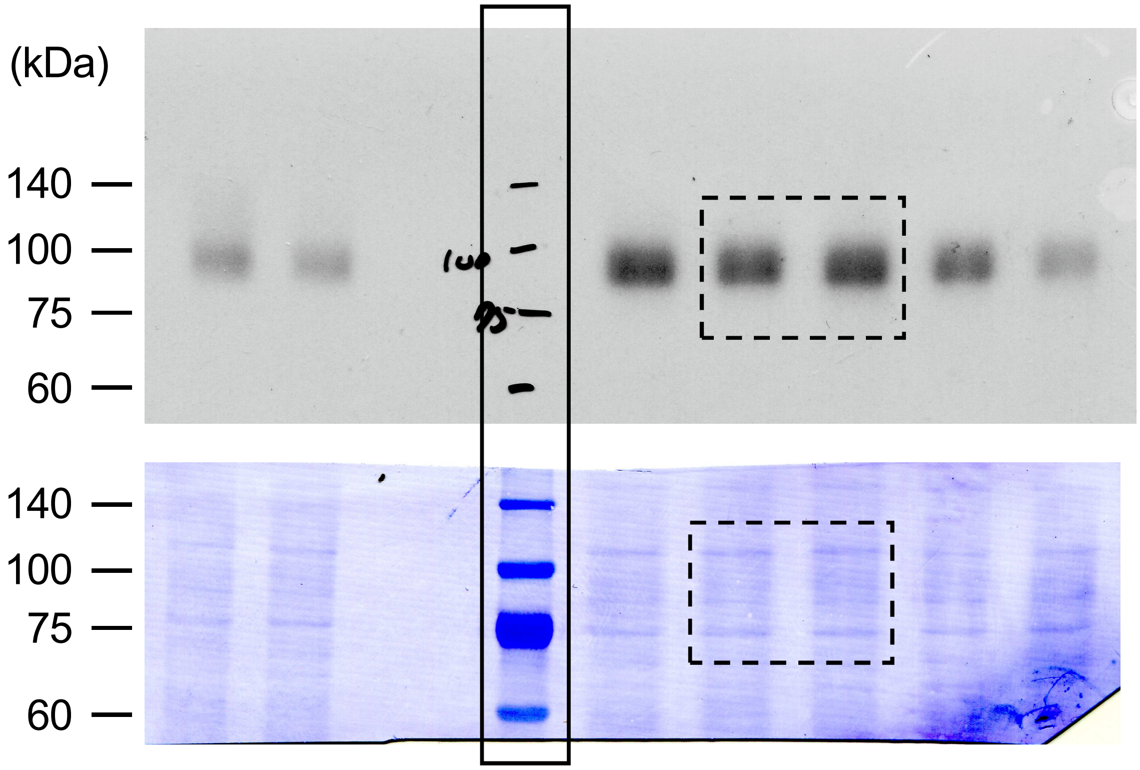

Supplementary Figure 9. Uncropped blot images of Supplementary Fig. 8. Dashed boxes indicate the areas used in Supplementary Fig. 8. Undashed box indicates the lane for size markers. The film in the upper side was obtained with the PVDF membrane in the lower panel.
